# Methodology to Derive Objective Screen-State from Smartphones: A SMART Platform Study

**DOI:** 10.3390/ijerph16132275

**Published:** 2019-06-27

**Authors:** Tarun Reddy Katapally, Luan Manh Chu

**Affiliations:** 1 Johnson Shoyama Graduate School of Public Policy, University of Regina, 2155 College Ave, Regina, SK S4M0A1, Canada; 2 Johnson Shoyama Graduate School of Public Policy, University of Saskatchewan, 101 Diefenbaker Pl, Saskatoon, SK S7N 5B8, Canada; 3 College of Medicine, Health Science Building, 107 Wiggins Road, University of Saskatchewan, Saskatoon, SK S7N 5E5, Canada; 4 Canadian Centre for Health and Safety in Agriculture, University of Saskatchewan, 104 Clinic Place, PO Box 23, Saskatoon, SK S7N 2Z4, Canada

**Keywords:** screen-state, screen time, smartphone, mobile health, digital epidemiology, citizen science

## Abstract

Time on screens (screen time) on multiple digital devices (computers, mobile phones, tablets, television screens, etc.) due to varied motivations (work, leisure, entertainment, gaming, etc.) has become an integral part of population behaviour. However, a significant evidence gap exists in screen time accumulated over ubiquitous mobile devices such as smartphones. This study aimed to develop an accurate, reliable and replicable methodology to derive objective screen time (i.e., screen-state) from all types of citizen-owned smartphones. A convenience sample of 538 adults (≥18 years) from two largest urban centres in Saskatchewan, Canada (Regina and Saskatoon) was recruited in 2017 and 2018. Participants used a custom-built smartphone application to provide objective and subjective data. A novel methodology was developed to derive objective screen-state, and these data were compared with subjective measures. The findings showed that objective screen-state from smartphones can be derived and assessed across a range of cut-points that take into consideration varied measurement errors. When objective measures were compared with subjective reporting, the results indicated that participants consistently underreported screen time. This study not only provides a methodology to derive objective screen-state from ubiquitous mobile devices such as smartphones but also emphasises the need to capture context via subjective measures.

## 1. Introduction

With the advent of the digital age, time on screens (screen time) on multiple digital devices (computer, mobile phone, tablet, television screens, etc.) due to varied motivations (work, leisure, entertainment, gaming, etc.) has become an integral part of population behaviour [1]. Screen time research has been widely incorporated across multiple disciplines, including public health, epidemiology and psychology, with emerging evidence suggesting that screen time is associated with health issues such as poor sleep [1] overweight/obesity [2,3], physical inactivity [4] and poor cognitive development [5]. However, these findings are not always consistent [1], and it is important to separate the construct of screen time across different behaviours (i.e., entertainment, video gaming, etc.) [6] and digital devices.

As screen time is predominantly assessed using self-report questionnaires that primarily focus on assessing television [7,8], video gaming, texting [9] and computer time [10,11,12,13,14], without taking into account the variation of these behaviours across different digital devices, there is a significant gap in evidence related to screen time accumulated over mobile devices, such as smartphones. Smartphones have become the truly ubiquitous devices, with some estimates projecting that there will be 6 billion smartphones in circulation by the year 2020 [15]. Thus, it is critical to understand not only the health impact of smartphone-based screen time accumulation, but also how smartphone-based screen time, i.e., smartphone usage, influences behaviours such as active living and dietary intake [16,17,18,19,20].

Traditional self-report and cross-sectional research designs have previously been used to quantify smartphone screen time [14,15]. Self-reporting of smartphone screen time is prone to significant measurement errors, with evidence indicating that self-reported findings are inconsistent [13]. Utilising ecological momentary assessments, Kobayashi and Boase [21] (2012) showed that smartphone users over report frequencies of communication usage on their devices. Similarly, Moreno et al. [22] (2012) found low correlation between ecological momentary assessment of Internet use and self-reported Internet use, to conclude that self-reported smartphone-based screen time via traditional validated surveys results in overestimation.

Thus, evidence indicates that existing self-report measures, especially for smartphone-based screen time, may result in significant bias due to the consistent dependence on this ubiquitous device. A novel approach is needed to collect real-time data with multiple time-stamped assessments over time to minimise recall bias and errors in over- and/or underestimation of smartphone use. Smartphones are equipped with built-in sensors that can objectively capture smartphone screen time (i.e., screen-state), and although one smartphone addiction study showed that self-report time is significantly lower than objectively measured smartphone use, to our knowledge [23], a replicable methodology to derive objective smartphone screen-state does not exist.

As part of the SMART Platform, a citizen science and mobile health initiative [24], this study aimed to develop an accurate, reliable, and replicable methodology to derive prospective objective screen-state usage from all types of citizen-owned smartphones functioning on both Android and iOS platforms. Moreover, this study also aimed to compare prospectively obtained objective screen-state with retrospectively obtained validated self-reported measures of screen time that were adapted to capture different user behaviours (Internet surfing, texting, etc.) on smartphones.

## 2. Materials and Methods

### 2.1. Study Design

**Figure 1:**
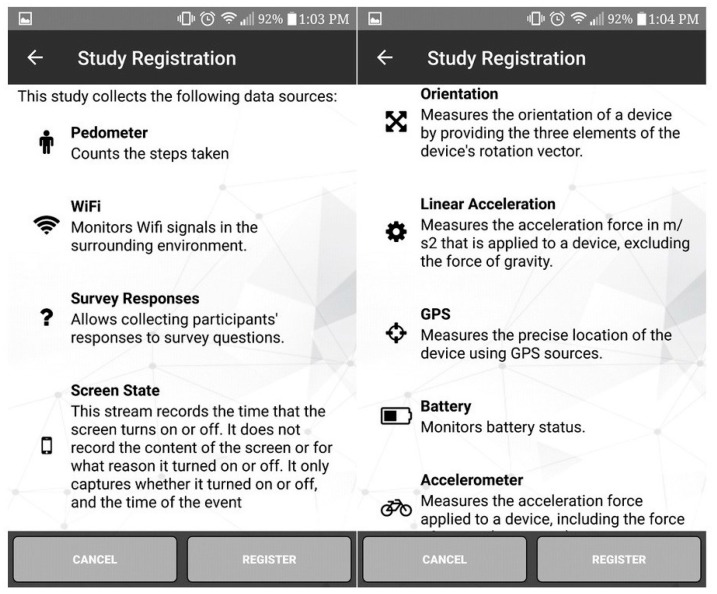
Objective and subjective measures of SMART Platform.

Data were obtained from the adult cohort of the SMART Platform (Regina, SK, Canada) and detailed description of methods, including recruitment and data collection strategy has been described in the methodology of the SMART Platform [25]. In brief, SMART Adults [24] is a prospective cohort study designed to obtain longitudinal data from a convenience sample of adults (≥18 years). The study was designed to capture data across different seasons (winter, spring, summer, and autumn) during 8 consecutive days in each cycle. All subjective (including screen time) and objective data (including screen-state) related to physical activity, sedentary behaviour, perception of environment, individual motivation, health outcomes, and eudaimonic well-being are obtained through citizen-owned smartphones during each cycle (Figure 1). The data that were used for this study were collected as part of pilots conducted between April 1 and May 31, 2017 and January 4 and March 31, 2018.

### 2.2. Study Recruitment and Participants

**Figure 2:**
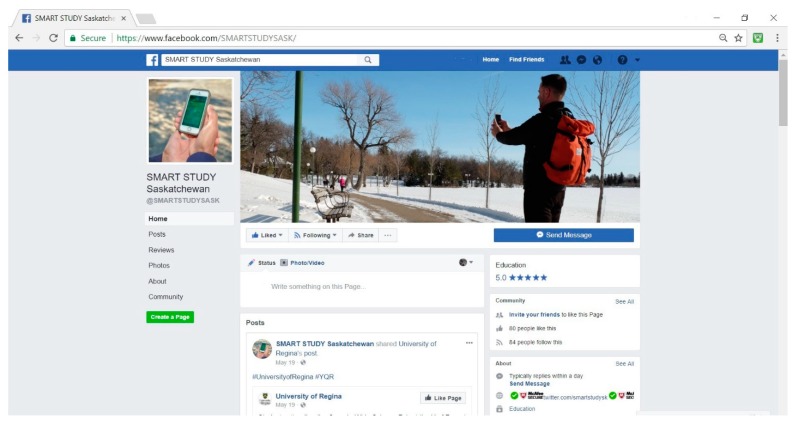
SMART Platform: Recruitment via social media.

Participants in the SMART Platform are termed as “citizen scientists” as they can engage with the researchers at all stages of the research process. Citizen scientists were recruited online through social media or in person from the universities of Regina and Saskatchewan, and community centres located in different neighbourhoods in Regina and Saskatoon to capture a representative sample (Figure 2). Citizen scientists were guided to download Ethica (Ethica Data Services Inc., Waterloo, ON, Canada), an epidemiological smartphone application (app), specifically adapted for the SMART Platform, which captures data through both Android and iOS platforms.

**Figure 3:**
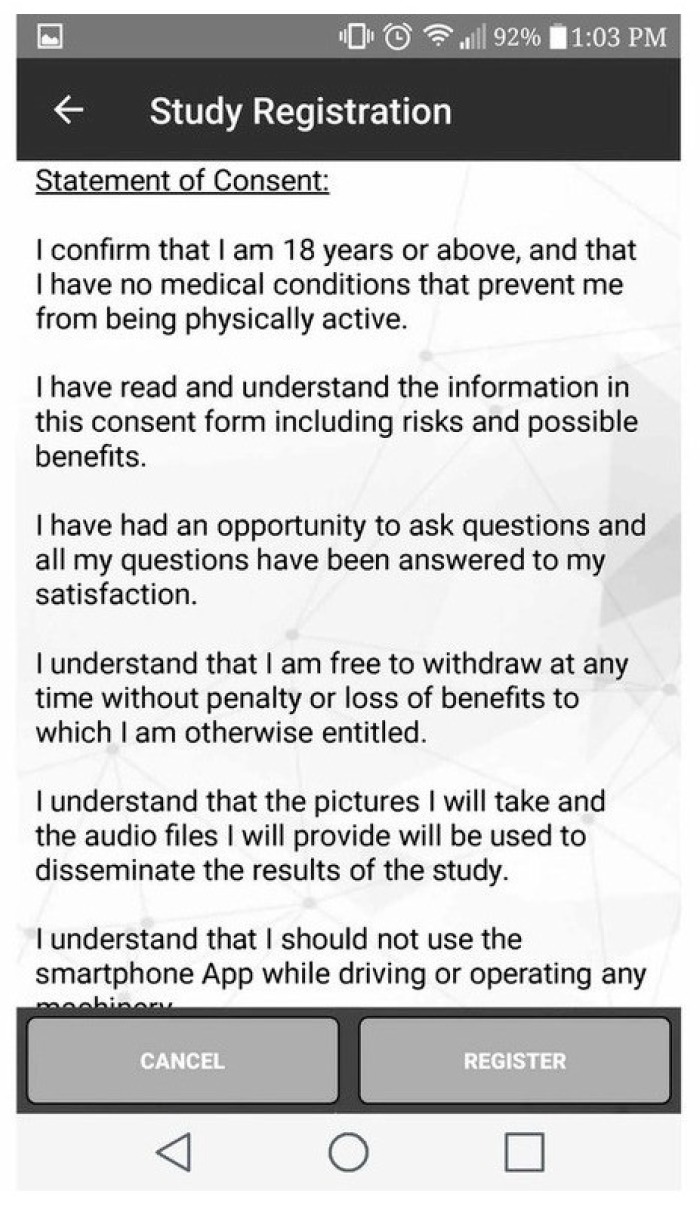
Informed consent provided via smartphone app.

All citizen scientists provided informed consent through the app (Figure 3) and confirmed their age (≥18 years) before being recruited. Ethics approval was obtained from the universities of Regina and Saskatchewan through a synchronised review protocol (REB # 2017-29).

### 2.3. Data Collection Tools

Objective time-stamped smartphone screen-state was captured through the screen-state sensor that recorded every ON and OFF screen notification, i.e., all smartphone screen time accumulated by participants over 8 consecutive days. Subjective screen time data were obtained via a modified sedentary behavior questionnaire [26] (University of California, San Diego version) that not only captured various motivations for screen time accumulation (gaming, texting, Internet surfing, etc.), but also varied devices (desktops, laptops, tablets) across which screen time can be accumulated:

1. On a typical WEEKDAY (from when you wake up until you go to bed), how much time do you spend watching TELEVISION?
2. On a typical WEEKDAY, how much time do you spend doing INTERNET SURFING (watching videos, reading news, etc.) or GENERAL WORK (office work, emails, paying bills, etc.) on a DESKTOP/LAPTOP/TABLET?
3. On a typical WEEKDAY, how much time do you spend doing INTERNET SURFING (watching videos, reading news, etc.) or GENERAL WORK (office work, emails, paying bills, etc.) on a SMARTPHONE?
4. On a typical WEEKDAY, how much time do you spend playing games on a DESKTOP/LAPTOP or TELEVISION SCREEN?
5. On a typical WEEKDAY, how much time do you spend playing games on a SMARTPHONE or a HANDHELD VIDEO GAME CONSOLE?
6. On a typical WEEKDAY, how much time do you spend TEXTING?
7. On a typical WEEKDAY, how much time do you spend SITTING and READING a paper-based BOOK/MAGAZINE?
8. On a typical WEEKDAY, how much time do you spend SITTING and READING an ELECTRONIC BOOK/MAGAZINE on a DESKTOP/LAPTOP/TABLET?
9. On a typical WEEKDAY, how much time do you spend SITTING and READING an ELECTRONIC BOOK/MAGAZINE on a SMARTPHONE?
10. On a typical WEEKDAY, how much time do you spend SITTING and LISTENING to MUSIC?
11. On a typical WEEKDAY, how much time do you spend SITTING and TALKING on the PHONE?
12. On a typical WEEKDAY, how much time do you spend SITTING and PLAYING a MUSICAL INSTRUMENT?
13. On a typical WEEKDAY, how much time do you spend SITTING and doing ARTWORK/ CRAFT
14. On a typical WEEKDAY, how much time do you spend DRIVING/RIDING in a CAR/BUS/ TRAIN, or any other mode of MOTORISED TRANSPORTATION?

Screen time accumulated over smartphones was derived using the questions relevant to smartphone screen time accumulation.

The following were the inclusion criteria:

1. Citizen scientists who provided screen-state data on at least 2 weekdays and 1 weekend day and completed that adapted sedentary behavior questionnaire [26].
2. Citizen scientists who did not turn off the app during the 8 days of study participation.

**Figure 4:**
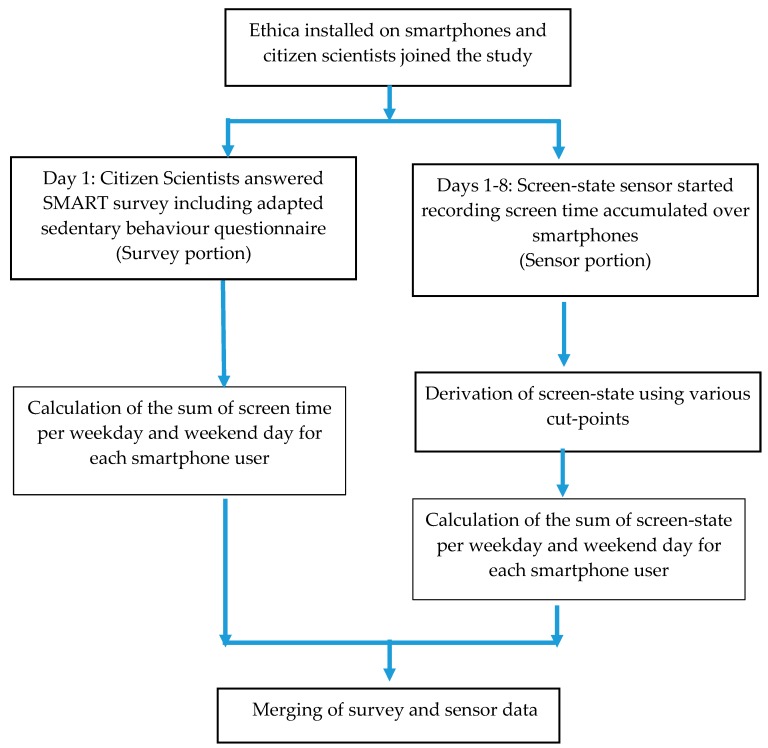
Stages of methodology development.

A description of the data derivation process to arrive at the final sample has been shown in Figure 4.

### 2.4. Methodology to Derive Objective Screen-State

A series of data processing techniques were applied to derive objective screen-state. After applying the inclusion criterion of deriving data only from those participants who provided data on at least 2 weekdays and 1 weekend day, screen-state was defined as “valid” when the smartphones went on to the “ON-state” (i.e., when the smartphone screens were activated). To take into account numerous real-life scenarios such as auto-notifications that result in activation of smartphone screens without participants actually viewing the screens, a series of “notification” cut-points for ON-state were developed, including 20, 15, 10, and 5 seconds.

Similarly, to account for invalid data due to smartphone errors that result in screens being in the ON-state longer than expected, “continuous usage” cut-points were developed with 6 thresholds: no-threshold, and 6, 5, 4, 3, 2 and 1 hours. These cut-points allowed the segregation of data by the longest duration of continuous usage of smartphones. Finally, another cut-point to account for potential invalid data due to long smartphone use was developed with two thresholds: usage of smartphones for less than or equal to 10 hours/day and more than 10 hours/day.

### 2.5. Statistical Analyses

Continuous estimates were reported as means with standard deviations (SD). After deriving objective screen-state data by implementing the above methodology, average screen-state time (in minutes) per weekdays and weekends for all users was calculated across a range of notification and continuous usage cut-points. Wilcoxon signed rank tests were conducted to compare the mean values of subjective screen time with mean values of objective screen-state across different cut points on both weekdays and weekend days. Correlation coefficients were calculated (Spearman’s rank or Pearson’s where applicable) to understand the correlation between subjective screen time and objective screen-state across different cut-points on both weekdays and weekend days. The Statistical Package for the Social Sciences for Windows v.24 software (IBM SPSS, Inc., Chicago, IL, USA) was used to conduct all analyses. The significance level was set to *p* < 0.05 (2-tailed).

## 3. Results

Out of 538 adult citizen scientists recruited in two different seasons (spring 2017 and winter 2019), 38 participants dropped out of the study. After applying the inclusion criterion of data being derived from only those participants who provided objective screen-state data on at least 2 weekdays and 1 weekend days and completed the adapted sedentary behavior questionnaire [26], the sample size for this study resulted in 54 adult citizen scientists (>18 years; males = 35.1% (*n* = 19); females = 64.9% (*n* = 35)).

Table 1, Table 2, Table 3 and Table 4 show results of the Wilcoxon signed rank tests comparing weekday and weekend mean duration of subjectively reported screen time and objectively derived screen-state (minutes per day) stratified by a series of “notification” and “continuous usage” cut-points. Table 1 and Table 2 show that on weekdays, although insignificant, irrespective of length of smartphone usage per day (>10 or ≤ 10 hours), overall participants underreported the screen time spent when compared to sensor-determined screen-state.

Similarly, on weekend days (Table 3 and Table 4), the over reporting trend for subjective screen time continued. When stratified by a series of “notification” and “continuous usage” cut-points, irrespective of length of smartphone usage per day (>10 or ≤10 hours), overall participants underreported the screen time spent when compared to sensor-determined screen-state, with significant differences being observed within the no-threshold category for continuous usage.

In terms of subjective screen time measurement, participants underreported usage more on the weekends in comparison with the weekdays, resulting in significant differences between objective screen-state and subjective screen time on the weekends. It must be noted that all significant results were observed within the no-threshold continuous usage cut-point category, irrespective of the range of the notification cut-points.

**Figure 5:**
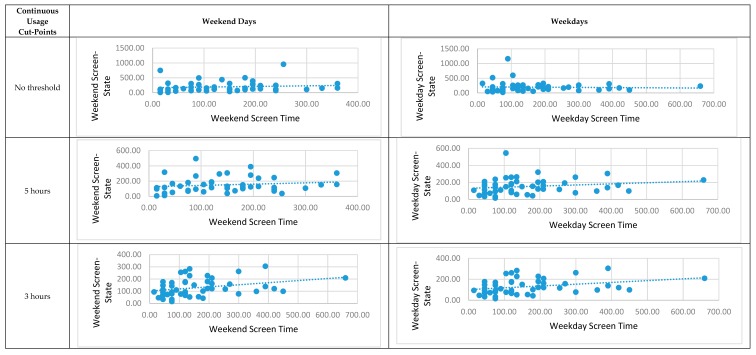

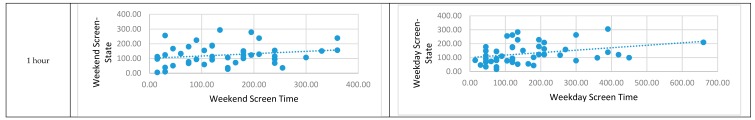
Correlation between objective screen-screen state with 20 second notification cut-point and subjective screen time across weekdays and weekend days.

Correlation analyses results are shown in Figure 5. In general, the correlation was either weak or not statistically significant between screen-state and screen time. For example, on weekdays, low statistically significant correlation was only found in “continuous usage” cut-points from 1 to 4 hours, and the overall trend was that when the “continuous usage” cut-points increased, the correlation coefficients decreased.

## 4. Discussion

The purpose of this study was to develop an accurate, reliable, and replicable methodology to derive objective screen-state usage from all types of citizen-owned smartphones and, within the same sample of participants, compare objective screen-state with validated self-reported measures of screen time that were adapted to capture different user behaviours (Internet surfing, texting, etc.) on smartphones.

After conducting a series of sensitivity analyses by testing “notification” and “continuous usage” cut-points to derive real-time screen-state, the findings showed that there are some statistically significant differences between objectively derived screen-state and subjectively reported screen time. The main results indicate that participants generally underreport their screen time compared to objective measures, both during weekdays and weekend days. These results were corroborated by correlation analyses, which showed low correlation between objective and subjective measures.

As all significant results were observed within the no-threshold continuous usage cut-point category, irrespective of the range of the notification cut-points, it shows that the difference between objective screen-state and subjective screen time exists even after taking into consideration real-life scenarios of participants receiving notifications on their smartphones throughout the day, with or without their knowledge. Moreover, these results also indicate that participants who are continuously using their smartphones for very long periods are significantly contributing to the difference between objective screen-state and subjective screen time.

Objective derivation of screen-state data from apps is not immune to measurement errors [27], which can occur when apps are actively turned on and off, the screen switches on without the users’ knowledge (i.e., automated notifications) or if multiple users use the same device. One of the inclusion criteria in this study was that participants had not switched off their apps during the data collection period. More importantly, a series of sensitivity analyses were conducted, which accounted for both automated notification and continuous usage of smartphones.

To our knowledge, this is the first study to develop a replicable methodology by examining optimal cut-points/thresholds to derive objective smartphone screen-state and compare it with a validated adapted survey that captured comprehensive screen time behaviours across multiple digital devices. Even with a small sample, and after taking into account multiple behaviours on smartphones (Internet surfing, texting, etc.), the results indicate underreporting via subjective measures, which challenges meagre evidence on smartphone screen time reporting via subjective measures.

This methodological study is part of the SMART Platform [24], which combines citizen science and community-based participatory research to engage with participants through their smartphones to understand various behaviours, including screen time accumulation across multiple digital devices. Thus, apart from the replicable potential of this methodology to derive objective screen-state from smartphones, the larger approach of combining citizen science and community-based participatory research could help to engage large populations across jurisdictions to understand patterns of smartphone-based screen time accumulation.

Another approach this study offers is linking objective and subjective measures of smartphone screen time to capture not only the accurate measurement of screen-state, but also essential context of behaviours (Internet surfing, texting, etc.). This approach can be used for both accurate surveillance of behaviours such as active living and dietary patterns, and as well as develop interventions using mobile technology [16,17,18,19,20]. Beyond the traditional linkage of objective and subjective measures, smartphone-based data collection also provides the opportunity to link big data obtained via sensors (such as accelerometers, pedometers, global positioning systems, Wi-Fi, and battery), with qualitative perception of citizen scientists through smartphone audio to accurately understand smartphone-based screen time behaviours.

More importantly, as the there is no indication that digital mobile device usage will decline in the future, and as these devices are being increasingly leveraged for mobile health applications [28,29,30], it is critical to understand both the positive and negative impacts of this technology on human health. This study is part of a mobile health platform that is utilising smartphones for both surveillance and interventions [25], and this novel methodology can be replicated in similar mobile health platforms to not only assess the link between smartphone usage and health outcomes but also inform interventions to minimize negative effects of smartphone usage, especially among those participants who are part of mobile health interventions.

### Limitations and Strengths

The sample size of the study was limited but still possessed enough power to depict statistically significant results, and we expect the findings to be reiterated when the methodology is replicated with a larger sample. With a larger sample, we expect to observe significant results across several cut-point categories, which would allow the modeling of health outcomes across different types of smartphone users, i.e., individuals who use smartphones rarely, intermittently, or constantly. This study is one of the first to measure smartphone screen time exposure prospectively [31], and this approach can help to track screen time in real-time, and longitudinally. Moreover, by adapting a validated recall survey, this study also serves as an example of modifying existing self-report measures to reflect technological advancements.

## 5. Conclusions

With smartphones becoming truly ubiquitous devices, it is paramount to understand the health effects of smartphone-based screen time accumulation, especially since screen time accumulated over different devices for different motivations might have a varied impact on health. This study advances a replicable methodology that can be used to prospectively derive objective screen-state and compare it with retrospectively derived subjective screen time from the same participants.

## Figures and Tables

**Table 1:** Objective screen-state vs. subjective screen time on weekdays (minutes/day) ^¥^.

*n*	Subjective Screen Time	Objective Screen-State					
		Continuous Usage Cut-Points	Notification Cut-Points				
			No Filter	5 Seconds	10 Seconds	15 Seconds	20 Seconds
	Mean		Mean (SD) (min/day)	Mean (SD) (min/day)	Mean (SD) (min/day)	Mean (SD) (min/day)	Mean (SD) (min/day)
47	165.00 (131.34)	6 hours	162.71 (98.52)	161.96 (98.17)	160.07 (98.42)	158.42 (98.25)	157.51 (98.10)
47	165.00 (131.34)	5 hours	160.43 (96.58)	159.68 (96.22)	157.79 (96.49)	156.13 (96.30)	155.21 (96.14)
47	165.00 (131.34)	4 hours	157.64 (94.82)	156.89 (94.43)	154.99 (94.67)	153.33 (94.43)	152.41 (94.24)
47	165.00 (131.34)	3 hours	156.06 (94.13)	155.31 (93.73)	153.42 (93.96)	151.76 (93.70)	150.84 (93.50)
45	166.25 (132.43)	2 hours	135.74 (73.08)	135.02 (72.66)	133.11 (72.65)	131.37 (71.97)	130.43 (71.61)
45	166.25 (132.43)	1 hours	134.94 (73.08)	134.22 (72.66)	132.30 (72.64)	130.56 (71.95)	129.62 (71.58)
49	165.00 (131.34)	All recorded time (No threshold)	200.47 (180.37)	199.73 (180.30)	197.86 (180.72)	196.26 (180.74)	195.34 (180.83)

**^¥^** Note: Comprising all screen-state records, including greater than 10 hours per day.

**Table 2:** Objective screen-state vs. subjective screen time on weekdays (minutes/day) ^¥^.

*n*	Subjective Screen Time	Objective Screen-State					
		Continuous Usage Cut-Points	Notification cut-points				
			No Filter	5 Seconds	10 Seconds	15 Seconds	20 Seconds
	Mean		Mean (SD) (min/day)	Mean (SD) (min/day)	Mean (SD) (min/day)	Mean (SD) (min/day)	Mean (SD) (min/day)
47	168.06 (142.02)	6 hours	154.65 (97.57)	153.95 (97.20)	154.19 (97.12)	154.19 (97.15)	153.31 (96.98)
47	168.06 (142.02)	5 hours	152.58 (95.62)	151.88 (95.24)	152.08 (95.18)	152.03 (95.20)	151.15 (95.02)
47	168.06 (142.02)	4 hours	150.05 (93.80)	149.34 (93.39)	149.49 (93.31)	149.40 (93.30)	148.51 (93.09)
47	168.06 (142.02)	3 hours	148.62 (93.05)	147.91 (92.63)	148.04 (92.57)	147.92 (92.55)	147.03 (92.33)
44	168.06 (142.02)	2 hours	147.87 (91.43)	147.16 (90.97)	147.27 (90.87)	147.12 (90.81)	146.23 (90.59)
45	169.25 (143.10)	1 hours	128.42 (73.30)	127.74 (72.85)	127.50 (72.53)	127.89 (70.98)	126.98 (70.61)
49	168.06 (142.02)	All recorded time (No threshold)	204.49 (186.42)	203.80 (186.37)	205.00 (187.28)	206.03 (188.23)	205.15 (188.34)

^¥^ Note: Excluding those with records greater than 10 hours per day.

**Table 3:** Objective screen-state vs. subjective screen time on weekend days (minutes/day) ^¥^.

*n*	Subjective Screen Time	Objective Screen-State					
		Continuous Usage Cut-Points	Notification Cut-Points				
			No Filter	5 Seconds	10 Seconds	15 Seconds	20 Seconds
	Mean		Mean (SD) (min/day)	Mean (SD) (min/day)	Mean (SD) (min/day)	Mean (SD) (min/day)	Mean (SD) (min/day)
47	144.18 (91.28)	6 hours	156.43 (100.09)	155.63 (99.97)	153.38 (100.63)	153.90 (104.19)	152.87 (104.14)
47	144.18 (91.28)	5 hours	154.84 (97.16)	154.04 (97.02)	151.78 (97.68)	152.30 (101.36)	151.27 (101.30)
47	144.18 (91.28)	4 hours	154.84 (97.16)	154.04 (97.02)	151.78 (97.68)	152.30 (101.36)	151.27 (101.30)
47	144.18 (91.28)	3 hours	154.84 (97.16)	154.04 (97.02)	151.78 (97.68)	152.30 (101.36)	151.27 (101.30)
45	145.31 (91.90)	2 hours	127.24 (71.95)	126.47 (71.73)	124.17 (72.08)	125.39 (69.89)	124.35 (69.71)
45	145.31 (91.90)	1 hours	127.24 (71.95)	126.47 (71.73)	124.17 (72.08)	125.39 (69.89)	124.35 (69.71)
49	144.18 (91.28)	All recorded time (No threshold)	200.37 * (177.58)	199.59 * (177.67)	197.37 ** (178.41)	197.8 ** (179.98)	196.81 ** (180.12)

* Significant at α=0.05 from paired *t*-tests; **borderline significant; **^¥^** Note: Comprising all screen-state records, including greater than 10 hours per day.

**Table 4:** Objective screen-state vs. subjective screen time on weekend days (minutes/day) ^¥^.

*n*	Subjective Screen Time	Objective Screen-State					
		Continuous Usage Cut-Points	Notification Cut-Points				
			No Filter	5 Seconds	10 Seconds	15 Seconds	20 Seconds
	Mean		Mean (SD) (min/day)	Mean (SD) (min/day)	Mean (SD) (min/day)	Mean (SD) (min/day)	Mean (SD) (min/day)
49	142.65 (90.22)	6 hours	153.00 (99.45)	152.22 (99.32)	150.02 (99.90)	150.51 (103.36)	149.52 (103.36)
49	142.65 (90.22)	5 hours	151.47 (96.57)	150.69 (96.42)	148.49 (97.01)	148.98 (100.57)	147.99 (100.49)
49	142.65 (90.22)	4 hours	151.47 (96.57)	150.69 (96.42)	148.49 (97.01)	148.98 (100.57)	147.99 (100.49)
49	142.65 (90.22)	3 hours	151.47 (96.57)	150.69 (96.42)	148.49 (97.01)	148.98 (100.57)	147.99 (100.49)
49	142.65 (90.22)	2 hours	147.32 (91.65)	146.55 (91.51)	144.36 (92.05)	144.85 (95.83)	143.87 (95.72)
47	143.70 (90.82)	1 hours	124.90 (71.31)	124.15 (71.08)	121.91 (71.37)	123.02 (69.28)	122.02 (69.07)
51	142.65 (90.22)	All recorded time (No threshold)	223.41 * (251.57)	222.64 * (251.70)	220.46 * (252.33)	220.85 * (253.27)	219.89 * (253.44)

* Significant at α = 0.05 from paired *t*-tests; ^¥^ Note: Excluding those with records greater than 10 hours per day.
